# Neuroticism facets and mortality risk in adulthood: A systematic review and narrative synthesis

**DOI:** 10.1016/j.jpsychores.2023.111500

**Published:** 2023-09-28

**Authors:** Marta Butler, Nicholas Turiano, Laura Buckley, Máire McGeehan, Páraic S. O’Súilleabháin

**Affiliations:** a Department of Psychology, University of Limerick, Limerick, Ireland; b Health Research Institute, University of Limerick, Limerick, Ireland; c Department of Psychology, West Virginia University, Morgantown, WA, USA

**Keywords:** Personality, Neuroticism, Facet, Mortality, Systematic review, Big five

## Abstract

**Objective:**

This systematic review sought to summarize comprehensively the research investigating the association between facets of neuroticism and mortality risk.

**Methods:**

A systematic review of prospective cohort studies utilizing rigorous reporting methods was conducted. Six electronic bibliographic databases, MEDLINE [Ovid], Embase, PsycINFO, CINAHL, Web of Science, and SCOPUS, were searched for eligible studies using keywords encompassing personality traits and mortality. Articles from inception to January 2023 were reviewed. The risk of bias was also assessed.

**Results:**

Six of the 2358 identified studies met the inclusion criteria for extraction. Included studies had 335,715 participants, of whom 3.23% died. Participants ages at baseline ranged from 20 to 102, and 54% were female. Five of the six studies reported statistically significant associations between facets of neuroticism and mortality risk. Several underlying facets were reported to be associated with an increased mortality risk, namely vulnerability, cynicism, pessimistic, anxious, and depressive facets. Inadequacy, and worried-vulnerable were reported as protective. One study reported protective effects for impulsiveness, but this was not observed in a further follow-up study.

**Conclusions:**

Various facets related to neuroticism are associated with an increased or decreased mortality risk. Encompassing all facets in a broad trait likely masks very important personality-health relations, which later impact longevity. Based on these findings, recommendations and future considerations are discussed.

## 1. Introduction

Neuroticism has long been of considerable interest within the context of health, with some even referring to it as a general “disease proneness” [21]. There has been a myriad of research implicating neuroticism as a psychological trait of profound public health significance [4,36,41,58]. Those with higher levels of neuroticism are more likely to rate their health as poor [26], experience poor mental health [24], smoke [44], report somatic complaints [45], be diagnosed with mental health disorders [35], display memory discrepancy [2], be at higher risk of Alzheimer’s disease and vascular dementia [61], and report psychological distress [22]. High neuroticism can also provide a pathway linking early-life socio-economic status to cognitive health later in life [55]. Regarding economic and societal costs, higher scores in neuroticism have been estimated to have a greater financial burden than substance abuse, mood disorders, and anxiety disorders [16].

Neuroticism has been hypothesized to be associated with health and mortality through different mechanisms (for review see [28]). For instance, health-risk behaviors such as sedentary behaviors and physical inactivity have been suggested as possible mechanisms [37]. Other possible pathways relate to heightened cardiovascular reactivity, hypothalamic-pituitary-adrenal (HPA) axis dysregulation, inflammation, and allostatic load across the lifespan [52,54,59,63,70]. In addition, individuals scoring high on neuroticism tend to expose themselves to more stressful situations and react with distressing emotions like anger [5,6,40]. As described above, this may occur through engaging in certain negative health-related behaviors. The combination of substance use, chronic stress, and altered cardiovascular function can be detrimental over the lifespan and lead to poor health outcomes and early mortality.

As a result of the broad spectrum of possible reported associations between neuroticism and health, studies began to examine the possibility that this trait may be associated with mortality risk [8]. Findings of studies on neuroticism and mortality appear somewhat inconsistent, at least comparably, to other personality traits such as conscientiousness [27,31,48]. Some studies have found that higher levels lead to increased mortality risk [42,46,56,66]; other studies found no significant link [1,14,30,32]; with other studies reporting that neuroticism appeared to have a protective effect against mortality risk [34,51,64].

While there are several potential reasons why these results may differ, it may be that different facets underlying neuroticism have different effects on mortality risk. In the Five-Factor Model [11], facets originate from a hierarchically organized structure and correlate with the general neuroticism trait. The need to study facets has long been recommended [9,10,11]. There are several ways to conceptualize personality traits and facets, resulting in various tools used to measure them. According to the Five Factor Model [10], the core facets of neuroticism are anxiety, angry hostility, depression, self-consciousness, impulsiveness, and vulnerability [11]. Measures such as the Minnesota Multiphasic Personality Inventory (MMPI; [17]), the NEO Five-Factor Inventory (NEO-FFI; [15]), the Eysenck Personality Questionnaire-Revised (EPQ-R; [19]) which is a shorter measure of neuroticism, or the International Personality Item Pool (IPIP; [25]), amongst others, have also been used to derive facets [23,29]. Individually examining facets may uncover possible underlying drivers of significant effects that an aggregate global measure of the broad domains of personality would otherwise mask. For instance, when completing a standardized measure of neuroticism, it is possible to achieve the same overall score for the broad domain of neuroticism, while the scores on the facets may differ considerably, thus suggesting potentially vastly different profiles [65].

Indeed, research examining neuroticism facets and health-related outcomes has grown. For instance, some research has found specific facets being associated with smoking behavior [33], alcohol consumption [38], gut microbiota [49], obesity [62], cardiovascular stress sensitization [47], and mental health [39]. Research also examined these facets with mortality risk; however, the extent of the literature and consistency across findings are unclear. For instance, some have reported specific facets linked to increased mortality risk [29] and others linked with protective effects [64]. As such, the extent of the literature and the confidence in the findings collectively are unclear.

Taken together, it is unclear what the individual drivers of the association between neuroticism and mortality risk may be. No rigorous systematic review on the relationship between neuroticism facets and mortality risk has been published to date. In this preregistered study, we systematically reviewed prospective cohort studies exploring the relationship between facets of neuroticism and all-cause mortality risk to advance the literature.

## 2. Methods

### 2.1. Protocol and registration

This review followed PRISMA guidelines for systematic reviews and meta-analyses. Accordingly, the review and protocol were preregistered in the PROSPERO database under the identification code CRD42022299283 on April 22, 2022.

### 2.2. Eligibility criteria

The primary objective was to select studies assessing the relation between neuroticism facets and all or cause-specific mortality. Therefore, only longitudinal cohort studies of samples during adulthood were included. Animal studies, child studies, intervention trials, or studies that did not report facets of neuroticism or mortality risk were excluded.

### 2.3. Information sources

English-language studies with no date restrictions were searched in Medline [Ovid], Embase, PsycINFO, CINAHL, Web of Science, and Scopus up until January 2023. The search strategy was designed with the support of a review-specialist librarian. The following terms and Boolean operators were applied: (“personality” OR personalit*) AND (“Neuroticism”) AND (“Mortality” OR “Mortality/trends” OR “Survival Analysis” OR “Longevity” OR Mortalit* OR death* OR dying OR dead OR longevit* OR surviv* OR “all-cause mortality” OR fatal* OR “survival analysis”). See Supplemental Material Table 3 for full search details. In addition, reference lists of the retrieved articles were screened to potentially identify more studies. Finally, the generated articles were de-duplicated through Clarivate Endnote (X9.3.3) and Covidence.

### 2.4. Study selection and data collection process

**Fig. 1.**
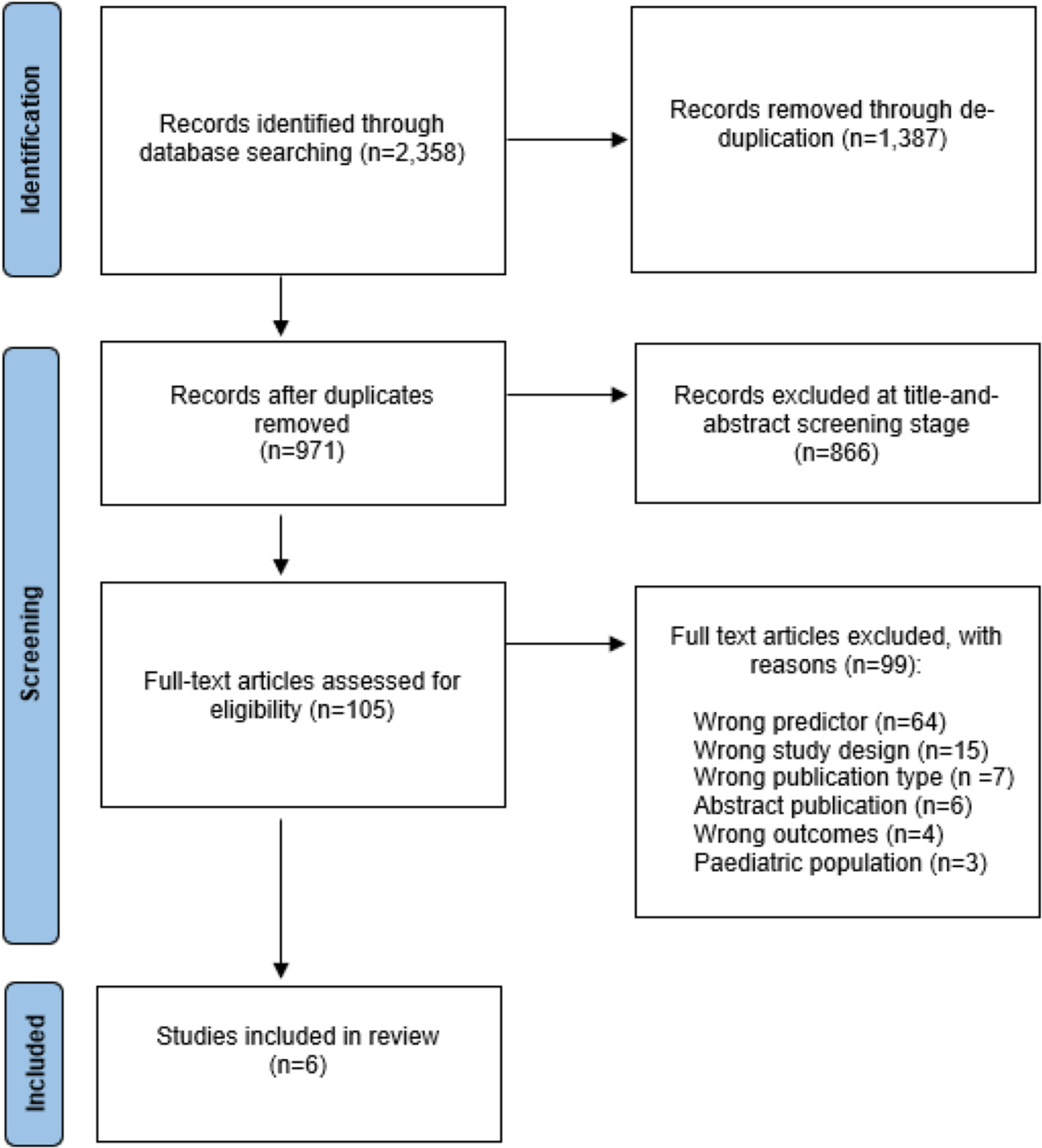
PRISMA flow diagram.

A Clarivate Endnote file of search results was exported to Covidence. First, two reviewers, blinded to each other’s assessment, screened articles based on title and abstract (97.84% agreement, Cohen’s κ = 0.89). Reasons for exclusion at the title and abstract screening stage included animal studies, pediatric studies, case studies, abstract publication, not relevant, and not retrievable. Next, full-text articles were assessed by two reviewers, MB and LB, for eligibility (99.04% agreement, Cohen’s κ = 0.90). Finally, the exclusion reason was recorded if a paper did not meet the inclusion criteria (Fig. 1). The reasons for exclusion at the full-text screening stage were the wrong predictor, study design, publication type, abstract publication, wrong outcomes, and pediatric population. Conflict about inclusion was resolved through discussion and group consensus.

The following data was extracted from included studies: general information about each study, study design and demographic information, methodological information, and outcomes variables. No assumptions or simplifications were made during extraction. If the reported data from the study were absent or ambiguous, the cell was filled in as not available (NA). A second reviewer (LB) checked all extracted information to ensure accuracy.

### 2.5. Assessment of risk of bias

The quality assessment of all included studies was conducted independently by two reviewers (MB and LB) using the Newcastle-Ottawa Scale (NOS) [68]. However, the exposure and outcome of interest items were not applicable due to the nature of the variables studied (personality and mortality). Hence, a modified scale version was utilized without the two selection items to adequately represent the study quality. Studies with NOS scores of 0–2, 3–5, and 6–7 were considered low, moderate, and high in quality, respectively (Supplemental Material Table 1). No studies were disadvantaged due to this change, as the excluded items did not apply to any of the studies. In addition, participant age was chosen as the most important confounding factor to examine comparability amongst studies. Conflict in quality assessment was resolved by reaching a group consensus through discussion.

### 2.6. Synthesis method

The method used to synthesize the results was narrative synthesis. This method was chosen to provide a comprehensive overview of the research on neuroticism facets and mortality risk. A meta-analysis was not feasible due to the high heterogeneity of data.

## 3. Results

### 3.1. Study selection

Of 2358 identified studies, 1387 were removed during de-duplication. The remaining 971 articles were screened in the title and abstract screening stage, of which 105 articles entered the full-text assessment for eligibility. Initially, five studies met the final inclusion criteria and were included in this review. During the review process a further publication was identified which had been excluded in the full-text screening. This manuscript [67] is now included in the data extraction and analysis.

### 3.2. Study characteristics

A total of six cohort studies from the United States (*K* = 5) and the United Kingdom (*K* = 1) were eligible for the systematic review (Table 1). One study [7] conducted an integrative analysis of three cohort studies. The participant cohorts used within the studies were Medicare (*K* = 2), Mayo Clinic cohort (*K* = 1), UK Biobank (*K* = 1), and the Baltimore Longitudinal Study of Aging (BLSA) (*K* = 1), Seattle Longitudinal Study (SLS) (*K* = 1), Long Beach Longitudinal Study (LBLS) (*K* = 1), and the Western Electric Study (*K* = 1). The overall number of participants across the six studies was 335,715, ranging from 597 to 321,456. The overall deaths in the samples were 3.23%, ranging from 1.4% to 90.9%. Females accounted for 52% of the sample, ranging from 0% to 75.9%.

### 3.3. Population

Grossardt et al. [29] utilized the Mayo Clinic Cohort Study of Personality and Aging. There were 7216 participants. This cohort is a geographically defined subset of a previously established MMPI, which included approximately 50,000 ambulatory patients from the United States who were referred to the Mayo Clinic for any medical reason with the exemption of acute illness, surgical procedure, psychiatric disorder, or hospitalization. The Mayo Clinic outpatients were administered the MMPI for research purposes between 1962 and 1965. The subset used in the study comprised participants located in a cluster of 72 counties within a 120-mile radius of Rochester, Minnesota. This restriction was applied to increase the reliability of the follow-up as it increases the likelihood that participants used the Mayo Clinic as a primary source of medical care following the MMPI testing. The exclusion criteria included participants referred to the Mayo Clinic for acute illness, surgical procedures, psychiatric disorder, or a period of hospitalization and participants residing outside of the 72 counties located approximately within a 120-mile radius of Rochester, Minnesota.

The participant sample utilized by Chapman et al. [7] comprised three United States Cohort Studies. The cohort studies were BLSA, SLS, and LBLS. The BLSA, initiated in 1958, is an aging cohort study of community-swelling volunteers residing predominantly in Baltimore, Maryland [20,57]. The BLSA began with a convenience sample of primarily white, college-educated men. Later recruitment has helped balance the sample by including minorities and, since 1978, women. Participants are generally healthy and highly educated. The SLS, initiated in 1956, is a cohort study focusing on cognitive aging [53]. All participants were members of an HMO, the Group Health Cooperative of Puget Sound, in the Seattle, Washington metropolitan area or were family members of these individuals. The Long Beach Longitudinal Study (LBLS) studies community-dwelling adults aged 30 and older [69]. It began in 1978. Participants are predominantly from Long Beach and Orange County, California. Early recruitment comprised members of a local HMO, Family Health Plan. After 2000, participants were recruited using direct mail with zip codes targeted to specific age groups.

Gale et al. [23] utilized the UK Biobank sample. The Biobank is a resource of 500,000 participants, established to identify determinants of disease in middle-aged and older people [60]. Between 2006 and 2010, community-dwelling adults aged 37–73 living in the UK were recruited.

The population used in the Weiss and Costa Jr [64] and Costa Jr et al. [14] studies were derived from a sample of 1444 community-dwelling adults aged between 65 and 100 years old. The participants were from upstate New York, West Virginia, and Ohio. The participants were part of the Medicare Primary and Consumer-Directed Care Demonstration, which is a randomized control trial of primary and consumer-directed care conducted by the Monroe County Long Term Care Program, Inc., in Rochester, New York, and the Center for Aging and Healthcare in West Virginia, Inc., in Parkersburg, West Virginia. All participants were enrolled in Medicare Part A and Part B, needing or receiving help with at least two activities of daily living (ADLs) or three instrumental activities of daily living (IADLs) and a recent history of significant use of healthcare services.

Weiss and Costa Jr [64] listed the following exclusions: participants failing a cognitive screen before completing the measures, participants who did not qualify for Medicare, participants who were not classified concerning eligibility, participants who did not provide educational data, participants who reported that they did not fill out the NEO-PI-R measures honestly and accurately. In 2014, Costa and colleagues outlined similar exclusion criteria for this cohort: participants not residing in the catchment area, participants who did not fill in the personality questionnaire, participants who missed more than 40 items on the NEO-PI-R, participants who did not pass NEO-PI-R validation screen, and participants who had missing data for any covariate.

Weiss et al. [67] utilized the Western Electric Study [50] participant sample [12,13]. The study recruited men employed by the Western Electric Company Hawthorne Works in Chicago, Illinois, for at least two years. The study was initiated in 1957 to identify factors associated with the risk of coronary heart disease.

### 3.4. Methodological characteristics

Three studies used the NEO-PI-R questionnaire, two used the MMPI, and one used the EPQ-R. Studies utilizing the NEO-PI-R reported a composite neuroticism score and scores for each of the six neuroticism facets. Gale et al. [23], who utilized the EPQ-R, reported a composite neuroticism score and generated two neuroticism facets, “anxious-tense” and “worried-vulnerable.” The study that utilized the MMPI [29] reported a composite neuroticism score and three neuroticism facets – pessimistic, anxious, and depressive. Weiss et al. [67] also utilized the MMPI and reported scores for neuroticism, cynicism, psychoticism, somatic complaints, and inadequacy. Two studies [23,29] introduced the facets simultaneously into the statistical models, and four studies introduced facets separately into the statistical models ([7,14]; Weiss & Costa, [64]; [67]).

### 3.5. Confounding variables

All six studies used covariates in the analyses. Age and gender were controlled for by all studies (*K* =6). Covariates included amongst studies were; education (*K* = 5), self-rated health (*K* = 4), smoking (*K* = 4), socio-economic status (*K* = 3), diagnosed conditions; CVD and diabetes (*K* = 3), and cancer, asthma, pulmonary disease (*K* = 1), Major Depressive Disorder (*K* = 3), alcohol consumption (*K* = 3), race/ethnicity (*K* = 2), physical attributes such as BMI and grip strength (*K* = 2), restrictions to activities of daily living (*K* = 2), health behaviors including exercise and diet (*K* = 1), systolic blood pressure (*K* = 1), serum cholesterol (*K* = 1), heart rate (*K* = 1) and reaction time (*K* = 1).

### 3.6. Statistical analysis

Five studies employed Cox’s proportional hazards, while [14] study utilized Accelerated Failure Time (AFT) modeling as an analysis method.

### 3.7. Mortality estimates

All findings are summarized in Supplemental Material Table 2. Grossardt et al. [29] reported that pessimistic, anxious, and depressive facets were associated with an increased risk of all-cause mortality in a primary analysis model adjusted for age and gender over a median follow-up time of 32 years. Secondary analyses included removing participants with less than a five-year follow-up, participants who died of unnatural causes (homicides, suicides, and accidents), and participants who scored in the top quartile of the depression scale at baseline. The effects for each facet were attenuated in size but remained statistically significant.

Chapman et al. [7] conducted separate survival analyses in the three samples using an identical model specification featuring a given facet. The models were adjusted for age, sex, education, and race/ethnicity. The final estimates were then used for integrative analysis between the three datasets, using a random-effect meta-analysis estimator to obtain pooled HRs and 95% CIs. The vulnerability facet was significantly associated with an increased risk of all-cause mortality over an average 19-year period, with an increase in the risk of 8% per standard deviation. Cause-specific mortality data was not reported. Gale et al. [23] found that higher scores on the “worry and vulnerability” facet were associated with lower mortality risk over a mean period of six years. The study reported HRs for all-cause and cause-specific mortality. Both models showed a significant association of higher scores on the worried-vulnerable facet with lower all-cause mortality. Model 1 adjusted for age and sex; each standard deviation increase was associated with a 12% reduction. Model 2 adjusted for other covariates (see Supplemental Material Table 2) and was associated with a 6% reduction per standard deviation increase. The worried-vulnerable facet had a significant effect on the following cause-specific mortalities in models adjusted for age and sex: cancer (7% reduction), CVD (16% reduction), and respiratory disease (16% reduction); however, the effects did not remain significant when adjusting for other covariates. There was no significant effect on death by external causes in either of the models. The anxious-tense facet was not significantly associated with all-cause or cause-specific mortality risk.

In the Weiss and Costa Jr [64] study, impulsiveness was associated with a reduced mortality risk over three years. Each standard deviation increase in impulsiveness was related to a 32% decline in all-cause mortality risk. This model included demographics, health status, and behavior covariates. Unadjusted effects were not reported in the study. Costa Jr et al. [14] was a follow-up study of Weiss and Costa Jr [64]. Using an 8-year mortality surveillance period, impulsiveness was no longer significantly associated with all-cause mortality. This study found no significant effects for any other neuroticism facets.

Weiss et al. [67] found the MMPI content factors of cynicism and inadequacy to be associated with long-term mortality in middle-aged men 45 years after baseline. Inadequacy was associated with lower all-cause mortality, and cynicism was associated with increased all-cause mortality.

## 4. Discussion

In this preregistered systematic review, we summarized and integrated the literature examining the association between neuroticism facets and mortality risk. Six articles were explored to determine if there was an association between neuroticism facets and all or any cause mortality. Overall, the findings were mixed. The research in the area is limited by scarcity, large variability between studies and methodological limitations. Two studies [7,29] reported facets as significant predictors of mortality: anxious, pessimistic, depressive, and vulnerability, while two studies [23,64] reported facets (impulsiveness and worried-vulnerable) with a protective effect, and one [14] reported no association. Weiss et al. [67] reported one facet (inadequacy) as protective and one (cynicism) as associated with an increased risk of mortality. Heterogeneity is present throughout the studies, including studies that utilized the same measures. Hereafter, we summarize and appraise our findings, consider the methodological differences, discuss the strengths and limitations, and provide recommendations for improving future studies investigating personality facets and mortality.

Considering impulsiveness, two studies used the same cohort with different follow-up periods of 3 and 8 years, providing a unique vantage point to the changing nature of the impact of personality on mortality, further influenced by the nature of the older cohort. The earlier study reveals a protective effect of impulsiveness. This effect appears counterintuitive, as impulsivity is often associated with risk-taking, thrill-seeking, and short-term gratification [10]. Hence, it may be hypothesized that impulsive individuals find it difficult to control cravings and urges, and they are expected to be more inclined to engage in health-harming behaviors such as overeating or smoking [3]. It is challenging to interpret why impulsiveness would be associated with a decreased risk of mortality. The authors suggest that a high score on impulsiveness in older people may reflect a slower rate of biological aging [64]. Considering the research implicating maladaptive coping styles in individuals with high impulsiveness, the authors posit that it is unlikely that the observed protective effect is not mediated behaviorally. The study calls for replication, and the follow-up study by Costa Jr et al. [14] reported no significant findings. An increased sample size and longer follow-up resulted in a significant effect no longer being observed. Importantly, power is an important consideration which is increased through increased deaths because of a longer follow-up period [67]. Considering that this is a cohort of older people, causes of death may move towards more distal and chronic causes, suggesting that there may be a point at which the effect of other factors (such as the presence of illness) has a more considerable influence on the impact of personality facets. Chapman et al. [7] did not find an association between impulsiveness and mortality. The authors hypothesize that impulsiveness may increase mortality risk, but perhaps earlier in life, via accidental deaths, which may be nullified by survivor bias.

Inadequacy, characterized by ‘shyness and feelings of incompetence when facing adversity’ [12], led to protective effects [67]. The authors highlight that it is challenging to interpret why this would be associated with a decreased risk of mortality, suggesting that individuals may be prone to being cautious and less likely to expose themselves to cumulative risk over time. It may also be possible that indirect pathways are present, and the authors state that it warrants further exploration. Cynicism was reported as associated with increased mortality risk [67]. The authors suggested that this appeared to be attributable primarily to deaths from cancer. Weiss and colleagues suggested several possible mechanisms, smoking, circulating levels of IL6, and behavioral factors related to health seeking and adherence [67].

The “worried-vulnerable” facet was associated with a reduced risk of death from all causes [23]. The authors speculate that worried individuals may be more likely to be vigilant in taking care of their health and may be more likely to be concerned with symptoms of ill health and seek help earlier, and this is supported by the findings that the facet was protective against death by cancer, CVD, respiratory disease, but no external, accidental causes. The authors discuss the evidence that higher neuroticism is linked with greater use of health services [16], which may mean that diseases are diagnosed and treated earlier, thus leading to greater survival. Vulnerable individuals may also elicit more help-seeking behaviors or be surrounded by others who care for them. Contradictory, Chapman et al. [7] found that vulnerability increased mortality risk, consistent across the three cohorts used within the analysis. Vulnerability, defined as a lack of perceived control and low mastery, may lead to chronic stress and worse health outcomes, as reported by the authors. The nature of this contradictory finding may well lie in the methodological differences between the studies, as different measures were used along with different covariates. It is important to note that the ‘anxious-tense’ and ‘worried-vulnerable’ [23] facets were obtained from exploratory bifactor analyses. In contrast to the facets for the NEO-PI-R, they are not correlated with the general neuroticism factor and, therefore, cannot be directly compared.

Grossardt et al. [29] reported a significant effect of pessimistic, anxious, and depressive personality facets on increased mortality risk. Pessimism accounted for most of the effects. The authors describe several possible causal underlying mechanisms for this association. Firstly, they suggest biological pathways that may lead to morbidity and mortality. Secondly, they suggest two types of behavioral pathways, one which may lead to poor self-care and self-neglect; and one which may modify disease progression following onset, such as the underutilization of healthcare. This study used a sizeable sample and a median follow-up time of 32 years. In addition, personality testing took place early in life, thus limiting threats from survivor bias. Chapman et al. [7] reported that higher scores on trait depression and anxiety showed a small association with survival in one of the cohorts (the BLSA) but showed an elevated risk in the other two cohorts, thus partially supporting the findings of Grossardt et al. [29]. Similarly, there are methodological differences between the studies, which may well contribute to the contradicting findings.

This systematic review posits several strengths, including conformity to the rigorous PRISMA method, preregistration, and transparency. It is the first systematic review to examine this association between neuroticism facets and mortality risk. Overall, the cohorts’ sizes are extensive and encompass long follow-ups with a primarily high average death percentage, except for the study utilizing the Biobank data. Several limitations to this systematic review were noted, the first of which lies within the construct of the facets. Three of the five studies utilized the NEO-PI-R, a “gold-standard” reliable and valid measure of neuroticism and facets. One study used the MMPI, deriving neuroticism facet scores from scores on the pessimistic, anxious, and depressive scales. One study used the EPQ-R and derived the neuroticism facets of “worry and vulnerability” and “anxious and tense.” The five studies thus yielded three distinct sets of facets. Facets derived by individual studies have no established reliability or validity; therefore, caution must be taken when examining the results. We acknowledge that combining results from studies that apply different measures of neuroticism facets increases inconsistency and weakens the generalizability. Furthermore, most studies in the systematic review represented the United States; only one of them, the United Kingdom, represented people living outside the geographic borders of North America. Finally, the generalizability of these results may be limited because the leading mortality risk factors can be different in high or middle-income countries compared to low-income countries [43]. Furthermore, it is probable that personality facets influence mortality differently in low-income countries.

Based on those limitations, we make the following recommendations to improve the quality and generalizability of future research in this area. First, future research would benefit from using reliable and valid measures of personality, which allow for the derivation of existing facets. Second, there are many statistical methods to examine finer grained aspects of neuroticism. The most common approach is to use mean facet scores. However, emerging evidence suggests that techniques such as bifactor modeling can decompose neuroticism into domain-level variance (trait neuroticism), facet-level variance (the unique variance attributed to each neuroticism facet) and incremental facet-level variance (the unique variance each facet has that is not explained by the neuroticism domain-level [18]. Such analyses may provide a more nuanced understanding of specific components of neuroticism are associated with health and longevity. Using more diverse samples in future studies would allow for greater representation. Replication of the above studies is required to validate the findings. Finally, a meta-analytic view of the neuroticism facets would benefit the field; however, more research in the area is needed.

Although mortality is an important outcome, it is crucial to also consider other outcomes, as they may provide greater specificity regarding the underlying disease process contributing to mortality risk. More long-term cohort studies are needed for reasons other than only capturing mortality. Future research needs to focus on identifying if certain facets have stronger or weaker effects at different points in the lifespan and whether protective or risk factors change across ages and populations. Identifying mortality-related facets will be crucial in understanding the complex relationship between neuroticism and health. Understanding how personality relates to health and mortality has the potential to improve health outcomes, inform clinical practice, and promote individual and population health.

## Supplementary Material

Supplementary Material

## Figures and Tables

**Table 1:** Characteristics of studies included in the systematic review.

Reference	Year	Country	Sample used	*N*	Deaths *N* (%)	Length to follow up* [range]	Mean age	Gender (% male)	Measure of N	Neuroticism facets

Weiss & Costa	2005	USA	Medicare Primary and Consumer-Directed Care Demonstration	597	108 (18.09)	Approx. three years [NA]	80.7	24.1	NEO-PI-R	anxiety, angry hostility, depression, self-consciousness, impulsiveness, vulnerability.
Grossardt et al.	2009	USA	Mayo Clinic Cohort Study of Personality and Aging	7080	4634 (65.5)	Median of 32.4 years [19 days to 44.3 years]	Median 48.1	48.7	MMPI	pessimistic, depressive, anxious
Costa et al.	2014	USA	Medicare Primary and Consumer-Directed Care Demonstration	597	367 (61.5)	Approx. 8 years [NA]	80.7	24.1	NEO-PI-R	anxiety, angry hostility, depression, self-consciousness, impulsiveness, vulnerability.
Gale et al.	2017	UK	UK Biobank	321,456	4497 (1.4)	Mean of 6.25 years [NA]	NA	45.96	EPQ-R	anxious-tense, worried-vulnerable
Chapman et al.	2020	USA	Baltimore Longitudinal Study of Aging, Seattle Longitudinal Study, Long Beach Longitudinal Study	4223	1248 (29.55)	Mean of 19.33 years [14 to 27 years]	63.45	46.17	NEO-PI-R	anxiety, angry hostility, depression, self-consciousness, impulsiveness, vulnerability.
Weiss et al.	2020	USA	Western Electric Study	1862	1693 (90.9)	45 years [NA]	47.3	100	MMPI	cynicism, psychoticism, somatic complaints, and inadequacy

* length to follow-up as reported in the study.

## Acronyms:

ADL: activities of daily living
AFT: Accelerated Failure Time
BMI: Body Mass Index
BLSA: Baltimore Longitudinal Study of Aging
CI: Confidence Interval
CVD: Cardiovascular Disease
EPQ-R: Eysenck Personality Questionnaire – Revised
DVT: Deep Vein Thrombosis
HMO: Health Maintenance Organization
HR: Hazard Ratio
LBLS: Long Beach Longitudinal Study
IADL: instrumental activities of daily living
IPIP: International Personality Item Pool
MDD: Major Depressive Disorder
MMPI: Minnesota Multiphasic Personality Inventory
NEO-FFI: NEO Five-Factor Inventory
NEO-PI-R: NEO Personality Inventory-Revised
NOS: Newcastle-Ottawa Scale
PRISMA: Preferred Reporting Items for Systematic Reviews and Meta-Analyses
RR: Risk Rations
SD: standard deviation
SLS: Seattle Longitudinal Study
