# Supplementary material for: Neuroticism facets and mortality risk in adulthood: A systematic review and narrative synthesis
Source: J Psychosom Res. Author manuscript; Available in PMC 2025 Apr 9. (PMC11979783; doi:10.1016/j.jpsychores.2023.111500)
Supplement: Supplementary Material [file NIHMS2061500-supplement-Supplementary_Material.pdf]

Supplementary Table 1: Risk of bias using modified Newcastle Ottawa scale

	Selection		Comparability		Outcome			Total	
**Study**	Representativeness of the exposed cohort	Ascertainment of exposure	Adjust for the most important risk factors **AGE**	Adjust for other risk factors	Assessment of outcome	Follow-up length	Loss to follow-up rate	Total quality score	Overall grade
**Weiss & Costa, 2005**	0	★	★	★	★	0	★	5	Moderate
**Grossardt et al., 2009**	0	0	0	★	★	★	★	4	Moderate
**Costa et al., 2014**	0	★	★	★	★	★	★	6	High
**Gale et al., 2017**	★	★	★	★	★	★	★	7	High
**Chapman et al., 2020**	★	0	★	★	★	★	★	6	Moderate
**Weiss et al., 2020**	0	0	★	★	★	★	★	5	Moderate

Supplementary Table 2: RRs of all-cause mortality of neuroticism facets

Reference	Covariates used in the models	Result HR (95% CI), *p*
Weiss & Costa, 2005	gender, age, educational achievement, smoking status, presence of CVD or diabetes, number of ADL or IASL restrictions, self-rated health, presence of MDD	N5:Impulsiveness 0.96 (0.94–0.99), *p* < .01
Weiss et al., 2020	1: age<br>2: age and behavioural and biomedical risk factors<br>3: age, education, behavioural and biomedical risk factors	Inadequacy<br>1. 0.96 (0.91-1.00), *p* = .12<br>2. 0.96 (0.92-1.01), *p* = .37<br>3. 0.89 (0.83-0.96), *p* = .013
		Psychoticism<br>1. 1.03 (0.98-1.08), *p* = .28<br>2. 1.02 (0.97-1.07), *p* = .52<br>3. 1.01 (0.94-1.07), *p* = .91
		Somatic complaints<br>1. 1.05 (1.00-1.10), *p* = .12<br>2. 1.02 (0.97-1.07), *p* = .52<br>3. 1.03 (0.97-1.10), *p* = .69
		Cynicism<br>1. 1.11 (1.06-1.16), *p* = <.001<br>2. 1.06 (1.01-1.12), *p* = .17<br>3. 1.10 (1.02-1.18), *p* = .049
Grossardt et al., 2009	gender, age	Pessimistic 1.32 (1.21-1.43), *p* < .001
		Depressive 1.36 (1.25-1.47), *p* < .001
		Anxious 1.30 (1.20-1.42), *p* < .001
Costa et al., 2014	gender, age, educational achievement, smoking status, presence of CVD or diabetes, number of ADL or IASL restrictions, self-rated health, presence of MDD	Anxiety 0.932 (0.832-1.945), *p* = .23
		Angry Hostility 0.988 (0.857-1.138), *p* = .86
		Depression 0.964, (0.853-1.089), *p* = .56

		Self-Consciousness 0.910, (0.806-1.027), *p* = .13
		Impulsiveness 0.913 (0.803-1.037), *p* = .16
		Vulnerability 0.923 (0.816-1.045), *p* = .20
Chapman et al., 2020	age, sex, education, and minority race/ethnicity	Anxiety 1.08 (0.98–1.20), *p* = .115
		Angry hostility 1.04 (0.97–1.13), *p* = .271
		Depression 1.13 (1–1.27), *p* = .055
		Self-consciousness 1.05 (0.99–1.13), *p* = .124
		Impulsiveness 1.02 (0.96–1.08), *p* = .473
		Vulnerability 1.08 (1.02–1.15), *p* = .005
Gale et al., 2017	1: age and sex<br>2: health behaviors – smoking, alcohol, exercise, fruit and vegetable consumption, physical attributes – BMI, forced expiratory volume, blood pressure, grip strength, reaction time, diagnosed disease (CVD/diabetes/cancer/asthma/ chronic lung disease, DVT, pulmonary embolism), SES, self-rated health	Anxious-tense<br>1. 1.00 (0.98-1.03), *p* = .905<br>2. 0.99 (0.96-1.03), *p* = .652
		Worried-vulnerable<br>1. 0.88 (0.86-0.91), *p* = <.001<br>2. 0.94 (0.90-0.97), *p* = <.001

Supplementary Table 3: Database final search strings

Database	Search #	Search Details	Results	Date
Medline (Pubmed)	4	(("Personality"[Mesh] OR personalit*[tiab]) AND ("Neuroticism"[Mesh] OR Neuroticism[tiab])) AND ("Mortality"[Mesh] OR "Mortality/trends"[Mesh] OR "Survival Analysis"[Mesh] OR "Longevity"[Mesh] OR Mortalit*[tiab] OR death*[tiab] OR dying[tiab] OR dead[tiab] OR longevit*[tiab] OR surviv*[tiab] OR "all-cause mortality" [tiab] OR fatal*[tiab] OR "survival analysis"[tiab])	357	12.01.2023
EMBASE	4	S1: ‘personality’/exp OR ‘personalit*’:ti,ab<br>S2: ‘neuroticism’:ti;ab<br>S3: ‘mortality’/exp OR ‘survival analysis’/exp OR ‘longevity’/exp OR Mortalit*:ti,ab OR death*:ti,ab OR dying:ti;ab OR dead:ti,ab OR longevit*:ti,ab OR surviv* or ‘all-cause mortality’:ti,ab OR fatal*:ti,ab or ‘survival analysis’:ti,ab	411	12.01.2023
PsycINFO (EBSCO)	4	(MH “personality” OR TI (personalit*) OR AB (personalit*)) AND (TI (neuroticism) OR AB (neuroticism)) AND (((MH "Mortality/TD/RF") OR (MH "Survival Analysis+")) OR (TI (mortalit* OR death* OR dying OR dead OR longevit* OR surviv* OR “survival analysis” OR "all-cause mortality" OR Fatal*) OR AB (mortalit* OR death* OR dying OR dead OR longevit* OR surviv* OR “survival analysis” OR "all-cause mortality" OR Fatal*)))	283	12.01.2023
CINAHL (EBSCO)	4	(MH “personality” OR TI (personalit*) OR AB (personalit*)) AND (TI (neuroticism) OR AB (neuroticism)) AND (((MH "Mortality/TD/RF") OR (MH "Survival Analysis+")) OR (TI (mortalit* OR death* OR dying OR dead OR longevit* OR surviv* OR “survival analysis” OR "all-cause mortality" OR Fatal*) OR AB (mortalit* OR death* OR dying OR dead OR longevit* OR surviv* OR “survival analysis” OR "all-cause mortality" OR Fatal*)))	141	12.01.2023

Web of Science	4	(((TS=(personalit*))) AND (TS=(neuroticism))) AND (TS=(Mortalit* OR "Survival Analysis" OR longevit* OR death* OR dying OR dead OR surviv* OR "all-cause mortality" OR fatal*))	699	12.01.2023
Scopus	1	TITLE-ABS-KEY (personalit*) AND TITLE-ABS-KEY (neuroticism) AND TITLE-ABS-KEY (mortalit* OR death* OR dying OR longevit* OR surviv* OR "survival analysis" OR "all-cause mortality" OR fatal*)	466	12.01.2023
